# Bifocal versus trifocal bone transport for the management of tibial bone defects caused by fracture-related infection: a meta-analysis

**DOI:** 10.1186/s13018-023-03636-5

**Published:** 2023-02-25

**Authors:** Kai Liu, Hongyan Zhang, Xiayimaierdan Maimaiti, Aihemaitijiang Yusufu

**Affiliations:** 1 Department of Trauma and Microreconstructive Surgery, The First Affiliated Hospital of Xinjiang Medical University, Urumqi, 830054 Xinjiang China; 2 The First Affiliated Hospital of Xinjiang Medical University, Urumqi, 830054 Xinjiang China

**Keywords:** Bone defects, Bone transport, Complications, Ilizarov method, Infection

## Abstract

**Background:**

The purpose of this meta-analysis was to compare the efficacy and outcomes of bifocal bone transport (BFT) and trifocal bone transport (TFT) for the treatment of tibial bone defects caused by fracture-related infection (FRI).

**Methods:**

The literature searches of Cochrane Library, Embase, Google Scholar databases, PubMed/Medline, and Web of Science for literature published up to September 20, 2022, were performed. The quality of the included studies was evaluated according to the MINORS scale. Patients were divided into the BFT group and the TFT group, depending on the site of the osteotomy. The demographic data, defect size (DS), external fixation time (EFT), external fixation index (EFI), bone and functional results, complications, and autologous bone grafting (ABG) were extracted and analyzed using the Review Manager software (version 5.3).

**Results:**

Five studies included 484 patients with tibial bone defects treated by bone transport investigated in this meta-analysis, with a mean bone defect of 9.3 cm. There were statistical differences in DS (MD =  − 2.38, 95% CI − 3.45 to − 1.32, *P* < 0.0001), EFT (MD = 103.44, 95% CI 60.11 to 146.77, *P* < 0.00001), and EFI (MD = 26.02, 95% CI 14.38 to 37.65, *P* < 0.00001) between BFT group and TFT group. There was no statistical difference in bone results (RR = 0.98, 95% CI 0.91 to 1.06, *P* = 0.67), functional results (RR = 0.94, 95% CI 0.82 to 1.07, *P* = 0.37), complications (OR = 1.57, 95% CI 0.59 to 4.14, *P* = 0.36), and ABG (RR = 1.2, 95% CI 0.78 to 1.84, *P* = 0.42) between two groups.

**Conclusions:**

TFT was a feasible and practical method in the treatment of massive tibial bone defects caused by FRI to receive shorter EFT and satisfactory bone and functional results.

**Supplementary Information:**

The online version contains supplementary material available at 10.1186/s13018-023-03636-5.

## Background

Bone defects caused by fracture-related infection (FRI) remain challenging orthopaedic problems for surgeons [1–3]. Over the past decades, bone defects of the lower extremities have been successfully treated with several protocols, including the Ilizarov technique [4], the Masquelet technique [5], vascularized autogenous bone grafting [6], etc. Bone transport, based on the Ilizarov technique, has gradually become the gold standard for the treatment of infectious bone defects since its advantages of radical debridement, and satisfactory outcomes of bone union and function recovery [7–9]. However, the limb functionality may be impaired by massive bone loss after previous excessive surgical procedures, including impaired vascular circulation, adjacent joint ankylosis and complex postoperative complications.

A study published by Borzunov showed the findings of multilevel bone transport for the management of extensive long bone defects and considered it may provide a solution for reducing the total treatment time [10]. Subsequently, many studies presented satisfactory results using bifocal bone transport (BFT) or trifocal bone transport (TFT) in the treatment of bone defects caused by FRI, including simplified surgical procedure, certainly reducing the total external fixation time, and fewer true complications [11, 12]. However, few meta-analyses or system review focus on the evaluation of indications and success rates of these two techniques. Therefore, the purpose of this study was to compare the efficacy and outcome of BFT and TFT for the treatment of tibial bone defects caused by FRI.

## Methods

### Literature search strategy

Comprehensive literature searches of Cochrane Library, Embase, Google Scholar databases, PubMed/Medline, and Web of Science for studies published up to September 20, 2022, were performed by our institutional library information specialist. Treatment strategies identified were as follows: Ilizarov technique, distraction osteogenesis, bifocal (single-level) bone transport, trifocal (double-level) bone transport, nonunion, and fracture-related infection.

The studies were included as follows: a series of more than ten patients, bone defect caused by FRI, treated by bone transport, and comparative trials reported by English. Publications reported non-original data (e.g., systematic reviews, meta-analyses, narrative reviews, commentaries, special technique), overlapped data, and non-English published studies were excluded.

### Data extraction

A comprehensive literature search was performed by two independent authors (KL and HYZ), and the quality of the methodology was assessed, which yielded 5 eligible articles [11–15]. Possible conflicts were resolved by a third reviewer. The following outcome variables were extracted for pooled analysis (Additional file 1):

1. General information of all studies, including study design and demographic data.
2. Clinical data of all studies, including defect size (DS), external fixation time (EFT), external fixation index (EFI), bone union time (BUT), bone and function outcomes, bone union rate, and complications.

### Quality assessment

The quality of the included studies was evaluated via the MINORS scale, which was considered suitable for surgical non-randomized controlled interventional studies [16]. MINORS quality evaluation table suggests that literature with a score less than 12 should not be included in Meta-analysis. In one study [11], EFI reported by months/cm was converted to days/cm.

### Statistical analysis

The Review Manager software (version 5.3, The Nordic Cochrane Centre, Copenhagen, Denmark) was utilized to perform statistical analysis and establish forest plots. Relative risk (RR) was used for dichotomous variables, and mean difference (MD) was applied for continuous variables as the combined statistic. The 95% confidence interval (95% CI) of variables was calculated and presented for pooled estimates. Heterogeneity among included studies was evaluated using the *I*^*2*^ statistics and Cochran's *Q* test. The random-effects model was applied when heterogeneity was significant (*P* < 0.05 or *I*^*2*^ > 50%), otherwise, the fixed-effects model was used. *P* < 0.05 was considered a statistically significant difference. The publication bias of the included studies was independently and graphically assessed for clinical outcomes using funnel plots.

## Results

### General population demographics

**Fig. 1:**
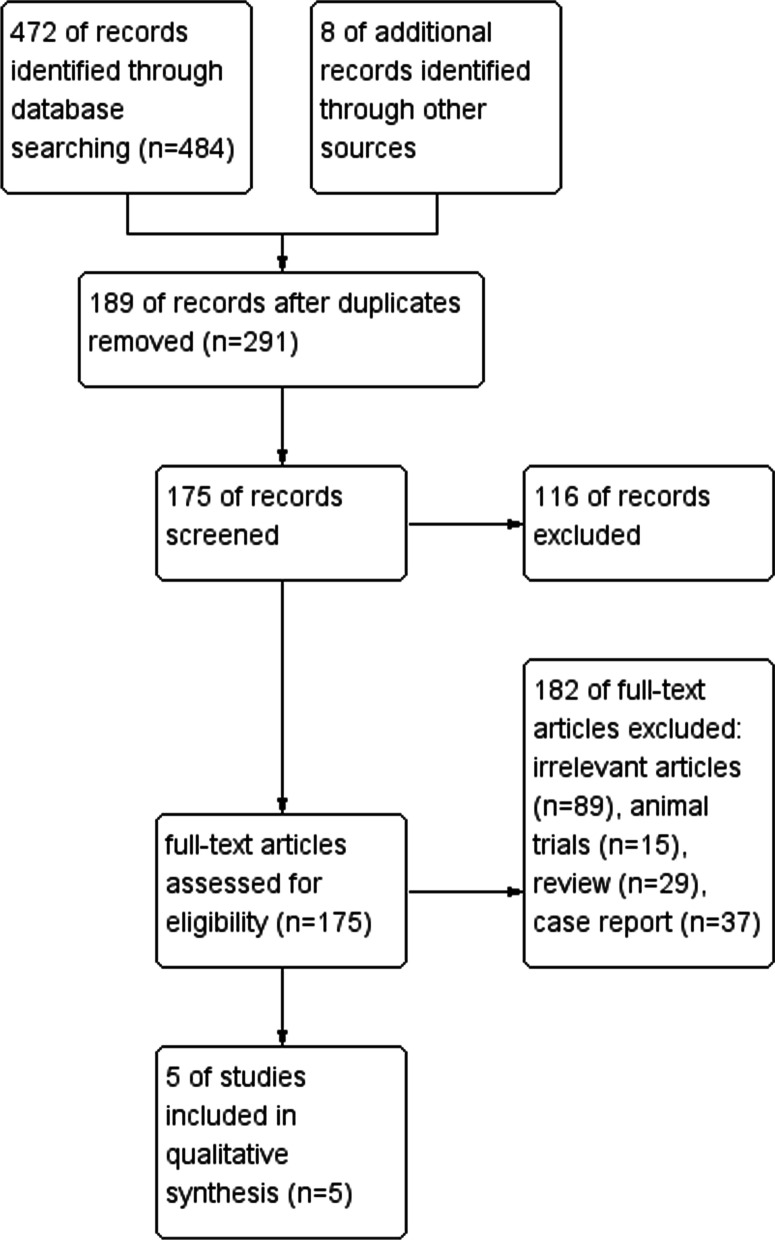
Inclusion flowchart

A total of 467 articles were excluded according to the inclusion and exclusion criteria after the initial selection, and 5 articles [11–15] were finally obtained. In these studies, 484 patients with tibial bone defects were treated by bone transport, with a mean bone defect of 9.3 cm. The document retrieval process was shown in Fig. 1. Studies describing only BTF or TFT were excluded to avoid heterogeneity. A quality assessment of included studies was performed using a MINORS (methodological index for non-randomized studies) checklist and presented in Table 1. Demographic data and clinical outcomes of the eligible studies were respectively presented in Tables 2, 3.

**Table 1:** Risk-of-bias assessment of the included studies according to the MINORS scale

Methodological items	Abulaiti 2022	Catagni 2019	Li 2020	Liu 2020	Yushan 2020
A clearly stated aim	2	2	2	2	2
Inclusion of consecutive patients	2	2	2	2	2
Prospective collection of data	0	0	0	1	0
Endpoints appropriate to the aim of the study	2	2	2	2	2
Unbiased assessment of the study endpoint	1	1	1	1	1
Follow-up period appropriate to the aim of the study	2	2	1	2	2
Loss to follow-up less than 5%	2	2	2	2	2
Prospective calculation of the study size	0	1	0	0	0
An adequate control group	1	1	1	1	1
Contemporary groups	2	2	2	2	2
Baseline equivalence of groups	1	2	2	2	1
Adequate statistical analyses	1	2	1	2	1
Total score	16	19	16	17	16

The items are scored 0 (not reported), 1 (reported but inadequate) or 2 (reported and adequate)

**Table 2:** Baseline characteristics of the included studies

Reference	Study design	Number of patients	Male/ female ratio	Age (year)	Follow-up time (month)
Abulaiti et al	RC	53	39/14	38.8(19–65)	nr
Catagni et al	RC	86	77/9	BFT, 43 (23 to 54)	45.6(26.4–108)
				TFT, 42 (33 to 51.5)	
Li et al	RC	26	20/6	40.4(22–56)	28.5(13–38)
Liu et al	R	282	243/39	40 (18–65)	≥ 24
Yushan et al	RC	37	28/9	40.1(18–57)	29.4(24–38)

BFT, bifocal bone transport; nr, not reported; R, retrospective; RC, retrospective comparison; TFT, trifocal bone transport

**Table 3:** Clinical outcomes of the included studies

Reference	Treatment technique	Number of patients	Infection nonunion (%)	DS (cm)	EFT (day)	EFI (day/cm)	Bone result (ASAMI, excellent and good)	Function result (ASAMI, excellent and good)	Complication (per patient)	ABG (%)	Bone union rate(%)
Abulaiti et al	BFT	32	10(31.2%)	7.8 ± 1.8	474.5 ± 103.2	60.8 ± 1.9	28	26	0.96(31/32)	4(12.5%)	32(100%)
	TFT	21	7(33.3%)	9.4 ± 1.5	328.0 ± 57.2	34.8 ± 2.1	19	18	0.52(11/21)	1(4.7%)	21(100%)
Catagni et al	BFT	45	16(35.5%)	12.5 ± 2.4	345.0 ± 54.1	27.6 ± 1.3	41	35	0.64(29/45)	16(35.5%)	45(100%)
	TFT	41	22(53.6%)	13.5 ± 2.5	261.0 ± 45.7	19.3 ± 1.1	38	33	0.46(21/45)	11(26.8%)	41(100%)
Li et al	BFT	13	13(100%)	7.2 ± 0.8	541.8 ± 44.7	75.7 ± 7.8	9	10	0.23(3/13)	4(30.7%)	13(100%)
	TFT	13	13(100%)	10.6 ± 2.3	381.3 ± 57.6	36.6 ± 6.0	11	12	0.3(4/13)	2(15.3%)	13(100%)
Liu et al	BFT	221	32(14.4%)	5.8 ± 1.6	385.1 ± 89.0	66.5 ± 8.5	182	106	0.3(68/221)	29(13.1%)	221(100%)
	TFT	61	9(14.7%)	9.1 ± 1.7	340.6 ± 52.1	38.0 ± 6.5	51	30	0.37(23/61)	9(14.7%)	61(100%)
Yushan et al	BFT	21	21(100%)	7.6 ± 2.3	299.9 ± 128.2	62.2 ± 24.6	19	17	0.8(17/21)	1(4.7%)	21(100%)
	TFT	16	16(100%)	10.3 ± 3.4	207.0 ± 40.4	32.9 ± 9.2	13	15	0.81(13/16)	1(6.2%)	16(100%)

ABG, autologous bone grafting; ASAMI, Association for the Study and Application of Methods of Ilizarov; BFT, bifocal bone transport; DS, defect size; EFI, external fixation index; EFT, external fixation time; nr, not reported; TFT, trifocal bone transport

### Defect size

**Fig. 2:**
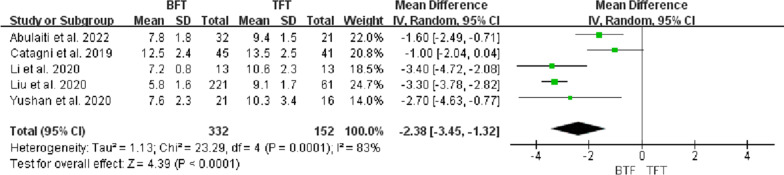
Comparison of DS between the BFT and TFT groups

Five studies [11–15] reported DS, including 484 patients. There were 332 patients in the BFT group and 152 patients in the TFT group. Heterogeneity analysis showed that there was significant statistical heterogeneity between these studies (*P* = 0.0001, *I*^*2*^ = 83%). Meta-analysis using a random-effects model presented that DS was significantly lower in the BFT group than in the TFT group (MD =  − 2.38, 95% CI − 3.45 to − 1.32, *P* < 0.0001). DS of the TFT group was greater than the BFT group, as shown in Fig. 2.

### External fixation time

**Fig. 3:**
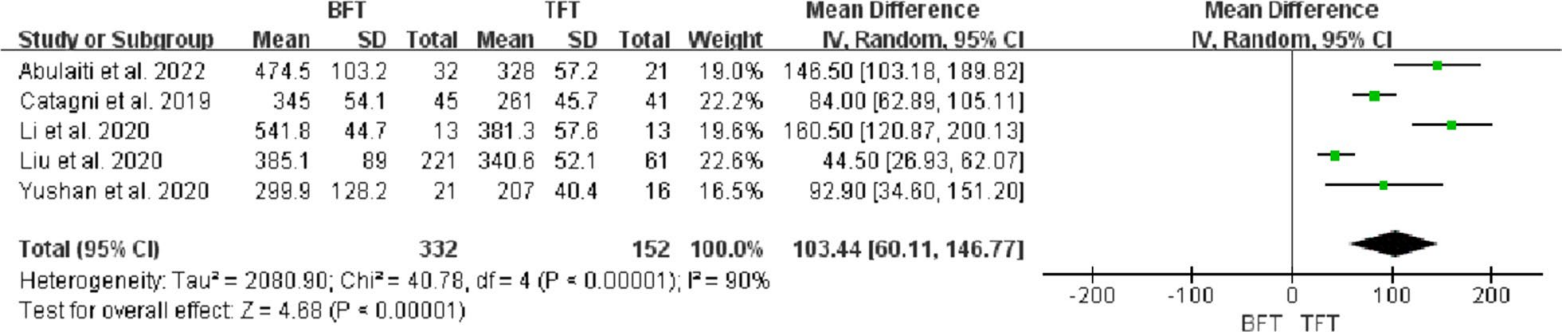
Comparison of EFT between the BFT and TFT groups

There was significant statistical heterogeneity in EFT (*P* < 0.00001, *I*^*2*^ = 90%) among these five studies [11–15]. The random-effects model analysis showed a statistical difference between the BFT and TFT groups (MD = 103.44, 95% CI 60.11 to 146.77, *P* < 0.00001). The results showed a statistical difference in EFT between the two groups, and the EFT of the TFT group was lower than the BFT group (Fig. 3).

### External fixation index

**Fig. 4:**
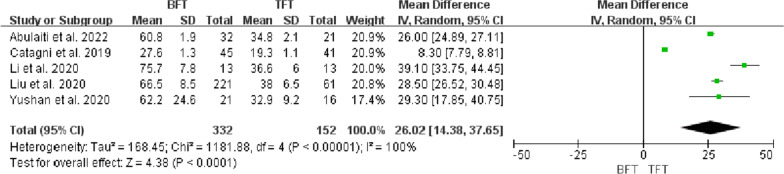
Comparison of EFI between the BFT and TFT groups

EFI was recorded by all five studies [11–15], and significant statistical heterogeneity was noticed (*P* < 0.00001, *I*^*2*^ = 100%). The random-effects model was utilized to analyse the data and there was a significant difference between the two groups (MD = 26.02, 95% CI 14.38 to 37.65, *P* < 0.00001). The results showed that the EFI of the TFT group was lower than the BFT group (Fig. 4).

### Bone results

**Fig. 5:**
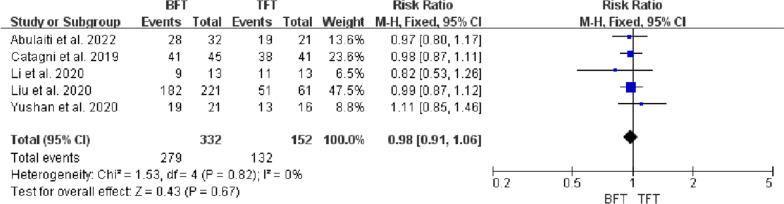
Comparison of bone results between the BFT and TFT groups

Five studies [11–15] reported bone results based on ASAMI criteria. Heterogeneity analysis showed no statistical heterogeneity among studies (*P* = 0.82, *I*^*2*^ = 0%). A fixed effects model was used for meta-analysis. The results showed that there was no significant difference in the excellent and good rate of bone results between the BFT group and TFT group (RR = 0.98, 95% CI 0.91 to 1.06, *P* = 0.67), indicating that the excellent and good rate of bone results was no statistical difference between two groups (Fig. 5).

### Functional results

**Fig. 6:**
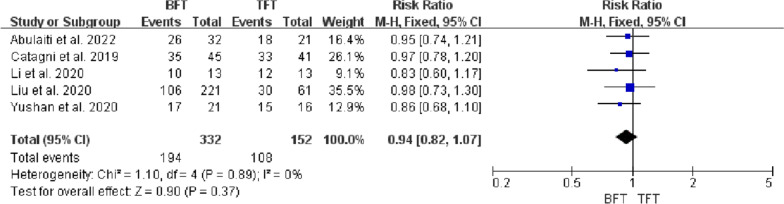
Comparison of functional results between the BFT and TFT groups

Functional results were documented in all five studies [11–15] based on ASAMI criteria. Heterogeneity analysis showed no statistical heterogeneity among studies (*P* = 0.89, *I*^*2*^ = 0%). Meta-analysis using a fixed-effects model showed that there was no significant difference in the excellent and good rate of functional results between the BFT group and TFT group (RR = 0.94, 95% CI 0.82 to 1.07, *P* = 0.37), indicating that the excellent and good rate of functional results was no statistical difference between two groups (Fig. 6).

### Complications

**Fig. 7:**
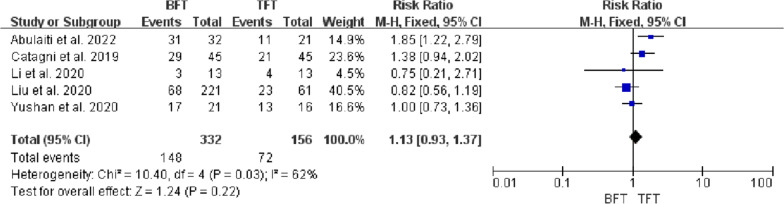
Comparison of postoperative complication between the BFT and TFT groups

Five studies [11–15] reported the rate of complications in two groups. Heterogeneity analysis showed that there was statistical heterogeneity among the studies (*P* = 0.01, *I*^*2*^ = 70%). A random-effects model was used for meta-analysis. Results showed that the TFT group had a lower complication rate than the BFT group. However, there was no statistical difference in the rate of true complication (OR = 1.57, 95% CI 0.59 to 4.14, *P* = 0.36), as shown in Fig. 7.

### Autologous bone grafting

**Fig. 8:**
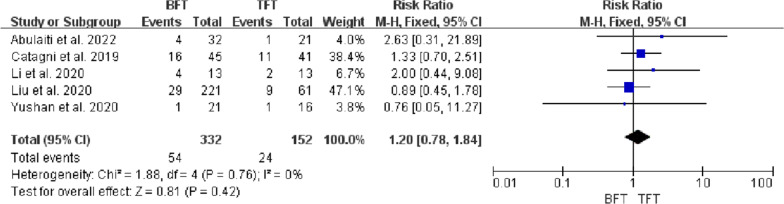
Comparison of autologous bone graft between the BFT and TFT groups

**Fig. 9:**
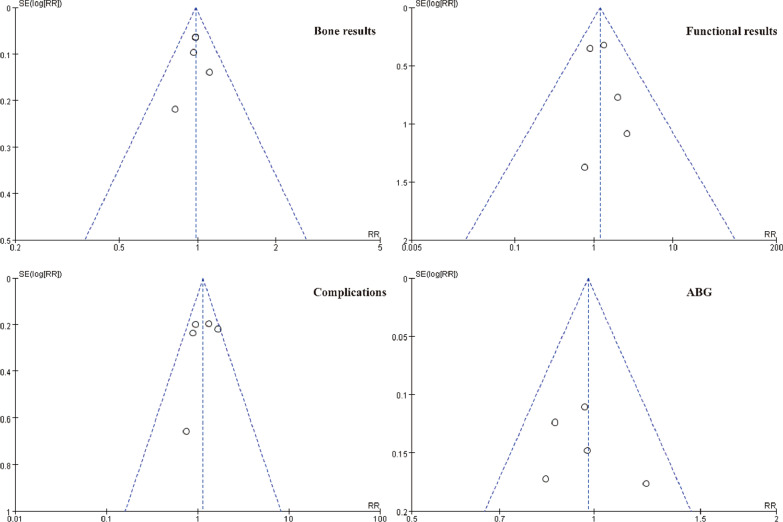
Funnel plot of the bone results, functional results, complications, and ABG between BFT and TFT groups

The rate of autologous bone grafting (ABG) was recorded in five studies [11–15], and no significant statistical heterogeneity was observed (*P* = 0.73, *I*^*2*^ = 0%). The fixed-effects model was applied and the statistical difference between the two groups was not noticed (RR = 1.2, 95% CI 0.78 to 1.84, *P* = 0.42), indicating that the rate of ABG was no statistical difference between the two groups (Fig. 8). The publication bias of bone results, functional results, complications and ABG were visually displayed using a funnel plot, which showed symmetrical distribution around the funnel plot, indicating low publication bias for those two groups (Fig. 9).

## Discussion

The bone defect caused by FRI usually involved both bone and soft tissue pathological conditions, which was a great challenge in the field of orthopaedics surgery [8, 9]. Treatment for bone defects caused by FRI was mainly characterized by infection control and bone regeneration [17]. The physiological function recovery of affected limbs depended on the functionality of callus formation and bone remodeling which both play a role of paramount importance. Bone transport had been reported as a practical method for the management of bone defects caused by FRI, since it could resolve both bone defect and soft tissue loss simultaneously via slowly distracting the transport bone segment to promote angiogenesis and osteogenesis, without the limitation of defect size [7, 10]. However, this technique also had the disadvantages of a long total treatment period, high risk of complications, and psychological burden of cumbersome appearance.

Borzunov et al. [10] described a method that multifocal bone transport using multilevel osteotomy could effectively reduce the EFT and EFI and receive satisfactory bone results, compared to bifocal bone transport. Subsequently, some studies on the trifocal bone transport technique had also reported that it had distinct advantages in the treatment of massive bone defects of the tibia [11, 12, 14]. Via previous articles [9, 10, 12] the distraction phase of multifocal bone transport was 2.5 times higher than bifocal bone transport, while the consolidation phase was reduced by 1.3 to 1.9 times. Further, Yushan et al. [18] initiatively found that tetrafocal and pentafocal bone transport could shorten the distraction phase, fasten bone regeneration, and reduce the associated complications. However, there were many comparative studies on bifocal and trifocal bone transport for the treatment of tibial bone defects, but no conclusions had been drawn from these results [11–15]. This study was the first meta-analysis addressing the issue.

DS usually determined the surgical strategy, which directly affected the condition of bone regeneration and functional recovery. Paley et al. [19] considered that bifocal bone transport was suggested to be used for DS > 10 cm. However, Robert et al. [20] suggested that trifocal bone transport should be considered when DS > 6 cm. Borzunov and Chevardin [21] suggested that poor bone regeneration might occur in the distraction area when bifocal bone transport was utilized to treat bone defects with DS > 5 cm or larger than 40% of the original segment. Liu et al. [13] thought that patients with DS > 6 cm treated by bifocal bone transport might lead to poor bone results and complications, including axial deviation, soft tissue incarceration and delayed union, which required additional surgical interventions and prolong EFT. In this study, there was a statistical difference in DS, EFT and EFI between the two groups (P < 0.05, Figs. 2, 3, 4), indicating that TFT was a reliable method for the management of bone defect > 6 cm.

The most common parameters of postoperative outcome evaluation using bone transport were EFT and EFI. EFT referred to the time spent until removing the external fixation, and EFI was defined as the ratio of EFT (days or months) to DS (cm). Despite the definition of BUT being still controversial, these were all essential indices for evaluating the quality of bone transport. Some scholars [9, 14] considered that BUT should be defined as the duration of consolidation without the distraction phase. Others [13, 15] believed that BUT was the total duration of bone union, which was similar to EFT. In this study, therefore, the value of EFI was uniformly defined as days/cm. Further, the mean EFI of the BFT group (range, 27.6–75.7 days/cm) was higher than the TFT group (range, 19.3–38 days/cm), which illustrated that TFT could significantly reduce the EFT and EFI.

As previously mentioned, bone transport using the Ilizarov technique, the ‘gold standard’ surgery, when the tibial bone defects caused by FRI, was to bridge the defect by promoting self-angiogenesis and osteogenesis. However, different results were yielded as the different distraction osteogenesis protocol and postoperative management after the surgery for most patients. ASAMI criteria were often adopted to assess bone and functional results in these five studies [8, 11–15]. The excellent and good rate of bone results in the TFT group (range, 81.2% to 92.6%) was higher than BFT group (range, 69.2% to 91.1%), and the excellent and good rate of functional results in the TFT group (range, 49.1% to 93.7%) was higher than the BFT group (range, 47.9% to 81.2%). However, there was no statistical difference in the excellent and good rate of bone results and functional results between the two groups (P > 0.05), which may be attributed to the limited number of literature. Although these two techniques were both practical in the treatment of tibial defects, more satisfactory outcomes of bone and functional results in the TFT group were received.

Pin tract infection was the most common complication associated with the utilization of external fixation in all studies [11–15, 19]. Further, the axial deviation was more likely to occur with the use of a unilateral external fixator. Complications were assessed according to Paley’s classification [19]. The incidence of complication in the BFT group (range, 23–96.8%) was higher than in the TFT group (range, 30.7–81.2%). Delayed union or nonunion at the docking site was the common complication in the management of massive bone defects, which usually required revision surgery. Despite cyclic distraction and compression (“accordion technique”) being feasible to salvage delayed union, ABG at the docking site after the distraction phase had still been advocated by some authors [8–10]. since a higher rate of the union. In this meta-analysis, the rate of revision surgery using ABG in the BFT group (range, 4.7–35.5%) was higher than in the TFT group (range, 4.7–26.8%). Hence, careful manipulation, detailed postoperative management and rehabilitation guidance played an important role in effectively preventing the occurrence of complications.

There were several potential limitations in this study. The included literature was non-randomized controlled studies with unclear methodological descriptions, which may result in a lower MINORS score. The analysis of outcome data was not performed by an independent investigator with blinding evaluation. The sample size of studies included in this Meta-analysis was small, and there was a lack of multicenter large-sample studies. There was a lack of methodology for outcome evaluation according to ethnicity, which may be a risk of bias. Therefore, further research may consider including prospective randomized controlled studies with large sizes of samples and blinding evaluation to avoid the potential risk of bias. Furthermore, it was also worth investigating the effect of combined techniques to promote bone regeneration to reduce the consolidation phase, including osteogenic factors, pharmacological agents, or bone formation-inducing proteins.

## Conclusion

This study was the first review of bifocal or trifocal bone transport for massive tibial bone defects caused by FRI, which identified the Ilizarov bone transport for the treatment of tibial bone defects caused by FRI. Overall published work showed a high bone union rate of 100% and a complication rate of 45.4% with no recurrence of infection. TFT was a feasible and practical method in the treatment of massive tibial bone defects caused by FRI to acquire shorter EFT and satisfactory bone and functional results. Radical debridement always played an essential role in controlling the infection. Pin tract infection was the most common complication with the utilization of external fixation. Dynamic technique and ABG were both effective methods for the management of docking site nonunion.

## Supplementary Information

**Additional file 1**. Appendix 1.

## Data Availability

The data sets generated and analyzed during the current study are not publicly available due to restrictions on ethical approvals involving patient data and anonymity but can be obtained from the corresponding author as reasonably required.
