# Supplementary material for: Bifocal versus trifocal bone transport for the management of tibial bone defects caused by fracture-related infection: a meta-analysis
Source: J Orthop Surg Res. 2023 Feb 25;18:140. doi: 10.1186/s13018-023-03636-5 (PMC9968413; doi:10.1186/s13018-023-03636-5)
Supplement: Supplementary file 1 — Additional file 1. Appendix 1. [file 13018_2023_3636_MOESM1_ESM.docx]

**Appendix 1**

**Search terms used for the individual databases**

**PubMed**

((("ilizarov technique"[MeSH Terms] OR ("ilizarov"[All Fields] AND "technique"[All Fields]) OR "ilizarov technique"[All Fields]) AND ("osteogenesis, distraction"[MeSH Terms] OR ("osteogenesis"[All Fields] AND "distraction"[All Fields]) OR "distraction osteogenesis"[All Fields] OR ("distraction"[All Fields] AND "osteogenesis"[All Fields]))) AND (("bone diseases, infection"[MeSH Terms] OR ("bone"[All Fields] AND "infection"[All Fields] AND "osteomyelitis"[All Fields]) OR " fracture-related infection "[All Fields] OR ("bone"[All Fields] AND "loss"[All Fields]) OR "bone loss"[All Fields]) OR defect[All Fields])) AND nonunion[All Fields]

**Cochrane Library**

("Ilizarov technique"):kw OR ("distraction osteogenesis"):kw OR ("bone transport"):kw AND ("bone defect"):kw OR ("bone loss"):ti,ab,kw AND ("infection") OR ("fracture-related infection"):ti,ab,kw

**Embase**

(‘ilizarov technique’/de OR ‘ilizarov ’/de OR ‘technique’):ab,ti) AND (‘infection’/exp OR ‘fracture-related infection’/de AND (distraction osteogenesis OR bone transport):ab,ti) AND (‘defect’/exp OR ‘bone defect’/de OR (bone loss):ab,ti) AND (‘observational study’/exp OR ‘cohort analysis’/exp OR ‘longitudinal study’/exp OR ‘retrospective study’/exp OR ‘prospective study’/exp OR ‘case control study’/de OR ‘cross-sectional study’/de OR ‘correlational study’/de OR ‘major clinical study’/de OR ‘multicenter study’/de OR ‘comparative study’):ab,ti)

**Google Scholar databases**

“infection/fracture-related infection” ilizarov technique/distraction osteogenesis/bone transport all intitle: “infection/fracture-related infection” ilizarov technique/distraction osteogenesis

**Web of Science**

((((((TS=(ilizarov technique)) OR TS=(distraction osteogenesis)) AND TS=(bone defect)) OR TS=(bone loss)) AND TS=(nonunion)) AND TS=(infection)) OR TS=(fracture-related infection)
